# Is percutaneous kyphoplasty safe and beneficial for patients aged 90 and over?

**DOI:** 10.1097/MD.0000000000030138

**Published:** 2022-08-19

**Authors:** Bo Yang, Yu Zhao, Yangxue Zhao

**Affiliations:** a Graduate School of Xi’an Medical University, Xi’an, China; b Department of Orthopaedics, the Ninth Hospital of Xi’an, Xi’an, China.

**Keywords:** advanced age, Osteoporotic vertebral compression fracture, Percutaneous Kyphoplasty, Secondary vertebral fracture

## Abstract

**Background:**

At present, to a large extent, we do not know the safety and benefits of minimally invasive surgery for elderly patients, especially the focus population of patients aged 90 and over.

**Methods:**

We analyzed 189 consecutive patients with osteoporotic vertebral compression fractures who underwent percutaneous kyphoplasty (PKP) between January 2018 and June 2021 at our institution. We divided them into the advanced age group aged 90 years or over (group A, n = 14) and the younger group under 90 years (group Y, n = 175). Clinical and complication indicators were evaluated and compared between the 2 groups.

**Results:**

A significant difference was observed in the procedure time, bleeding volume, and bone mineral density between the 2 groups during an average follow-up of 22 months. However, no significant difference was revealed in clinical and complication indicators between the 2 groups, and the pain and activity function of the 2 groups were significantly improved compared with those before PKP.

**Conclusions:**

Apparently, our results show that PKP is safe and beneficial for patients aged 90 years or older.

## 1. Introduction

Scientists around the world have been facing a huge challenge, that is, population aging. In the future, this problem will continue to attract people’s attention, especially in China, the world’s most populous country.^[1,2]^ Therefore, the health information of the elderly in China is closely monitored at all times. As a common disease of the elderly immobilization, the incidence rate of Osteoporotic Vertebral Compression Fractures (OVCFs) has increased significantly, which has affected >200 million people worldwide.^[3,4]^ Chronic back pain and activity limitation are the main clinical manifestations of OVCFs, which seriously affect the daily life of middle-aged and elderly people and reduce the quality of life. And the cost of treatment and care for this disease is high for the patient’s family.^[5]^

In the 1990s, Deramomd et al^[6]^performed minimally invasive surgery for OVCFs patients, which is different from traditional internal fixation, setting off a trend of minimally invasive treatment for OVCF patients. Bone cement injected into the fractured vertebral body can relieve pain and avoid long-term bed rest. However, direct high-pressure injection of bone cement has a high probability of leakage, and the correction of kyphosis is not significant.^[7,8]^ Fortunately, before bone cement is injected into the fractured vertebral body, an expandable balloon is used to pre form the cavity in the vertebral body, which can not only reduce the leakage of bone cement but also reduce the kyphosis angle of the spine.^[9]^ Therefore, percutaneous kyphoplasty (PKP) is frequently operated by orthopedics doctors because of its significant advantages.

Percutaneous vertebral augmentation significantly reduces the risk of death in OVCFs patients has been identified. In a study of >2 million patients, patients with OVCFs who underwent percutaneous vertebral augmentation were 22% less likely to die within 10 years after treatment than those who underwent nonsurgical treatment.^[10]^ In recent years, PKP has been proved to be safe and effective in the treatment of OVCFs patients, mainly manifested in immediate pain relief, kyphosis correction, and improvement of quality of life and wellbeing.^[11]^ Minimally invasive surgery has a simple process, so it has a wide range of patient selection. At present, to a large extent, we do not know the safety and benefits of minimally invasive surgery for elderly patients, especially the focus population of patients aged 90 and over.

We found a blank in this research field, so we studied whether patients aged 90 and over are suitable for minimally invasive surgery. The research on advanced age patients will further promote the development of minimally invasive surgery. After many reflections, we retrospectively analyzed the safety and sense of benefit of PKP in OVCFs patients aged 90 and over.

## 2. Materials and Methods

### 2.1. Study design

This well-designed retrospective study was conducted in our institution. Taking the age of 90 as the critical point, we divided the patients into the advanced age group and the younger group. By comparing the clinical and complication indexes between the 2 groups, we verified the theme of this paper, that is, whether the bone cement augmentation in advanced age patients is safe and beneficial. Therefore, patients who performed PKP in our institution and met the inclusion criteria were studied.

### 2.2. Patients

According to our careful statistics, from January 2018 to June 2021, 254 patients with OVCFs received PKP treatment in this institution. However, patients included in our study must meet the following criteria: diagnosis of osteoporotic vertebral compression fractures; the fracture was operated on within 1 month; there is only 1 fracture of thoracolumbar vertebral body; bone cement was injected into the vertebral body through bilateral pedicle; and continuous follow-up lasted at least 3 months. Ineligible patients are due to meeting the following criteria: old vertebrae fractures; symptoms of nerve damage were found; multiple vertebral fractures; unilateral bone cement injection was used; and follow-up data were not available.

### 2.3. PKP: technical considerations

The patient was placed on the operating table in the prone position. First, the vertebral body for operation is clearly located under the guidance of C-arm machine. And then routine preoperative disinfection and towel laying local anesthesia were performed. After completing the basic operation before puncture, we put the 11-gauge puncture needle into the appropriate position of the vertebral body under fluorescence guidance, that is, the lateral position of the X-ray is located in the front third of the vertebral body and the positive position is located in the central area of the vertebral body. The kyphoplasty balloon was placed in the vertebral body along the trajectory of the puncture needle, and then the balloon was slowly expanded. Under fluoroscopy, bone cement was injected into the cavity formed by balloon expansion. The endpoint of the operation was the uniform distribution of bone cement in the vertebral body or the infiltration of bone cement outside the vertebral body. All operation steps were completed by the same experienced orthopedic surgeon.

### 2.4 Study observation parameters

Visual analog scale (VAS) score and Oswestry Disability Index (ODI) are often used in the study as our clinical effect observation indicators. The former means that higher the score, the more unbearable the pain. And the latter means that the higher the score, the more unable daily activities are to be carried out. The above indexes were recorded 72 hours after operation. The leakage of bone cement is often asymptomatic and has no impact on the life of patients, so the secondary vertebral fracture after operation is taken as the observation index of complications. Eligible patients have been monitored by us during follow-up in order to find discomfort at the first time and prove the occurrence of secondary vertebral fracture. All data have been confirmed many times to ensure accurate research.

### 2.5. Statistical analysis

All collected data are divided into continuous data and categorical data. All continuous variables are first tested for normality. Then categorical data between 2 groups were analyzed using chi-square test and continuous data conforming to normal distribution between 2 groups were compared using the Student *t* test. The statistically significant difference was identified where *P* < .05 with hypothesis testing using a 2-tailed test of significance. All steps of data analysis are carried out using SPSS 18.0 software.

## 3. Results

### 3.1. Patient characteristics

**Figure 1.**
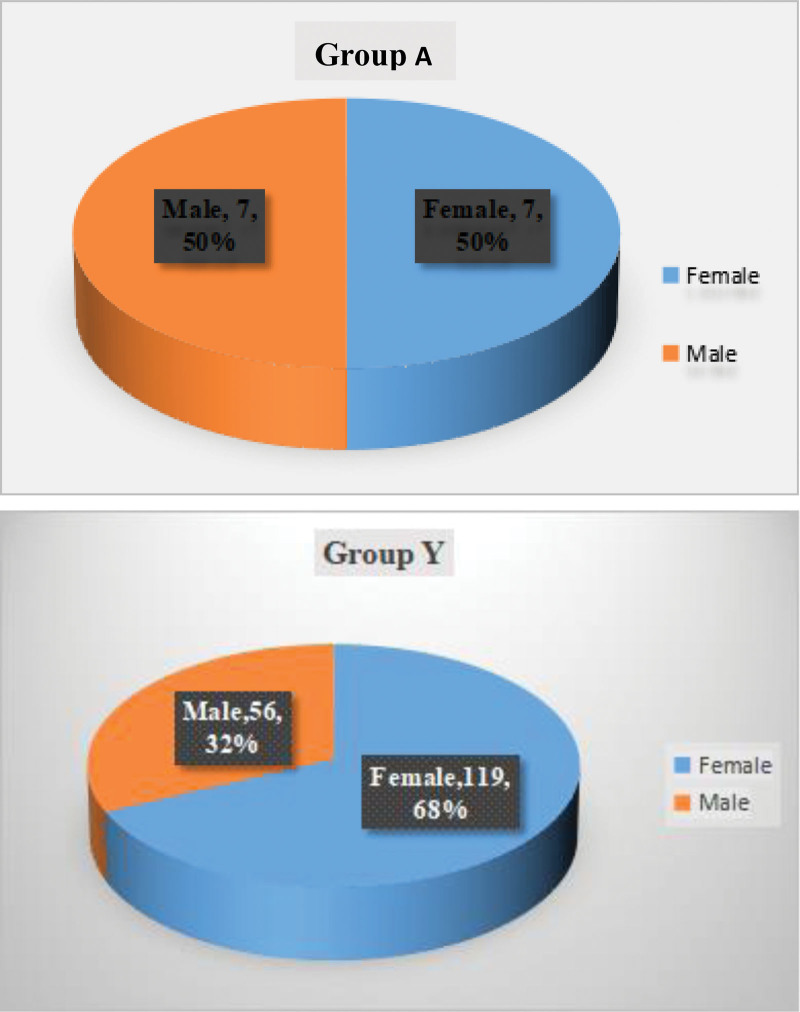
The pie chart distribution briefly summarized the basic composition information of patients in group A and group Y, and there was no difference in gender composition ratio between the 2 groups.

**Figure 2.**
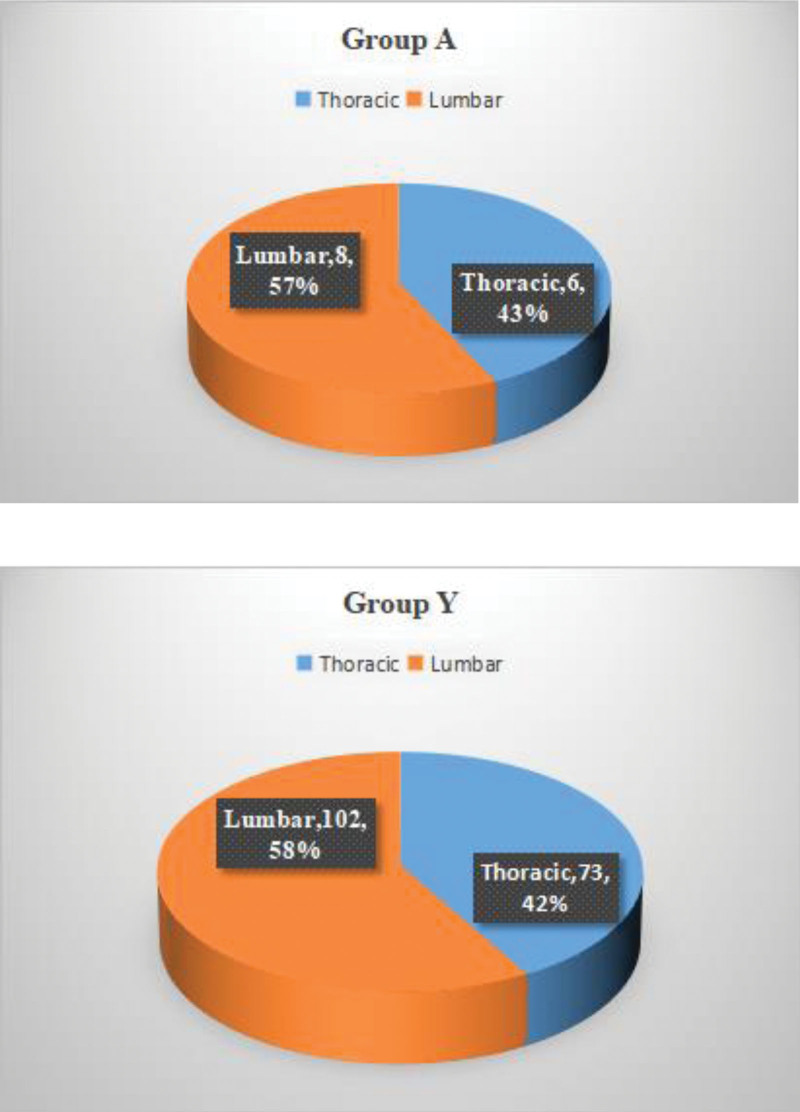
There was no significant difference in the surgical region between group A and group Y.

Two hundred fifty-four patients (93 males and 161 females) with OVCFs received PKP treatment in our medical institution from January 2018 to June 2021. However, 9 patients were excluded due to unilateral injection of bone cement, 14 patients had old vertebral fractures, and 37 patients had >1 fracture in the thoracolumbar segment. Finally, only 189 eligible patients were included in this study, 14 patients with aged 90 and over were included in group A, and the remaining 175 patients were included in group Y (Fig. 1). The fracture sites of the 2 groups were located in the common thoracolumbar vertebrae (Fig. 2). The background characteristics of the patients in the group Y and group A was summarized in Table 1. The average age of patients in group A was 93.36 years old, which was significantly different from that of patients in group Y was 74.41 years old. There were no identified differences in gender composition, BMI, bone cement injection volume, and follow-up time between the 2 groups. However, it is worth pondering that the differences in bone mineral density, bleeding volume, and procedure time were revealed.

**Table 1:** General information.

General information	Group A (n = 14)	Group Y (n = 175)	*P* value
Age (yr), mean ± SD	93.36 ± 2.82	72.41 ± 8.30	.000
BMI, mean ± SD	22.39 ± 3.13	22.98 ± 2.59	.420
BMD, T-score, mean ± SD	–3.78 ± 0.16	–3.10 ± 0.26	.000
Volume (mL), mean ± SD	5.14 ± 1.28	5.22 ± 1.04	.801
Bleeding (mL), mean ± SD	24.64 ± 11.00	16.03 ± 6.14	.004
Time (min), mean ± SD	35.71 ± 6.46	47.57 ± 7.56	.000
Follow-up (mo), mean ± SD	16.36 ± 14.29	21.98 ± 11.38	.083

BMD = bone mineral density, BMI = body mass index, SD = standard deviation.

### 3.2. Clinical outcomes

**Figure 3.**
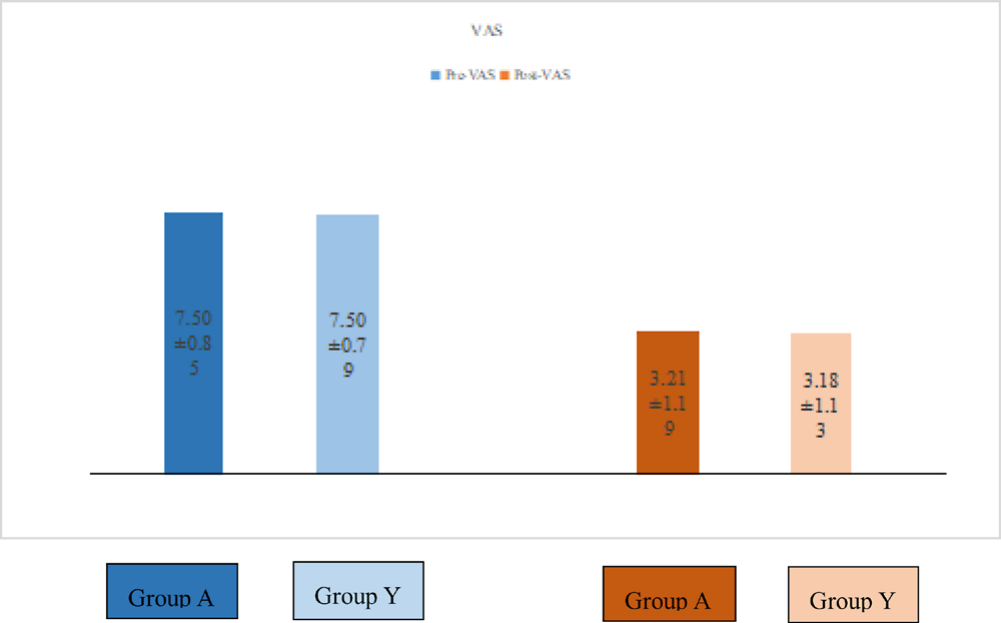
Pain outcomes scores (VAS) of the 2 groups in preoperation and postoperation, statistically significant improvements were identified (*P* < .05). VAS = visual analog scale.

**Figure 4.**
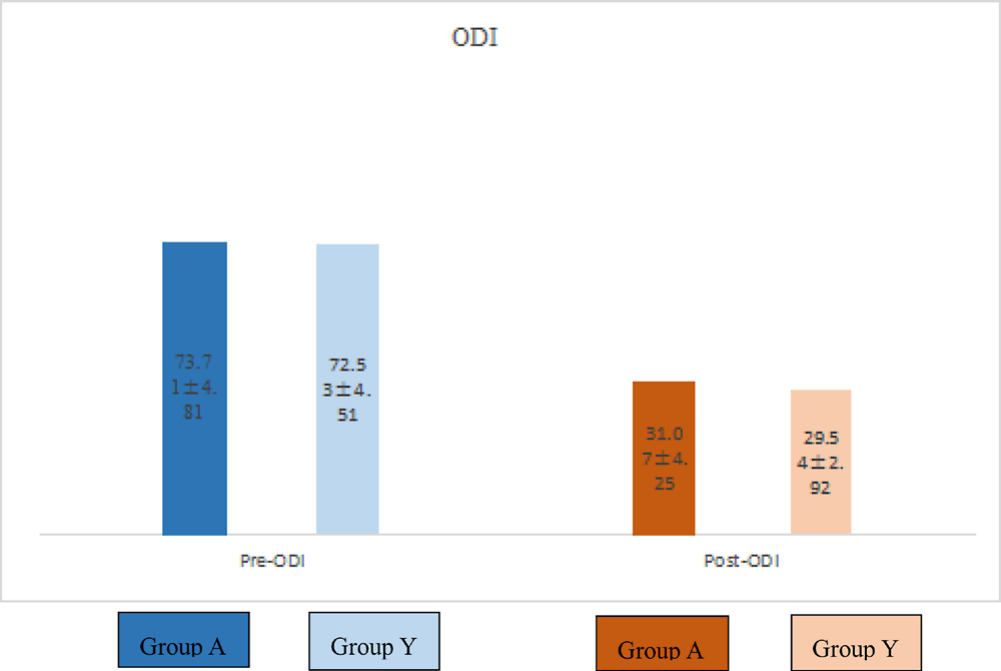
ODI of the 2 groups in preoperation and postoperation, statistically significant improvements were identified (*P* < .05). ODI = Oswestry Disability Index.

VAS score and ODI are often used in the study. We retrospectively collected VAS pain scores and ODI scores of all patients before operation; the average score of group A was 7.50 and that of group Y was 7.50, which was not the recognized difference. Similarly, no difference was distinguished in ODI score, which was 73.71 in group A and 72.53 in group Y. When we recorded VAS pain scores and ODI scores 72 hours after operation, we found that both were significantly reduced, indicating that the patient’s pain and activity function were significantly improved (Figs. 3 and 4).

### 3.3. Complications

**Figure 5.**
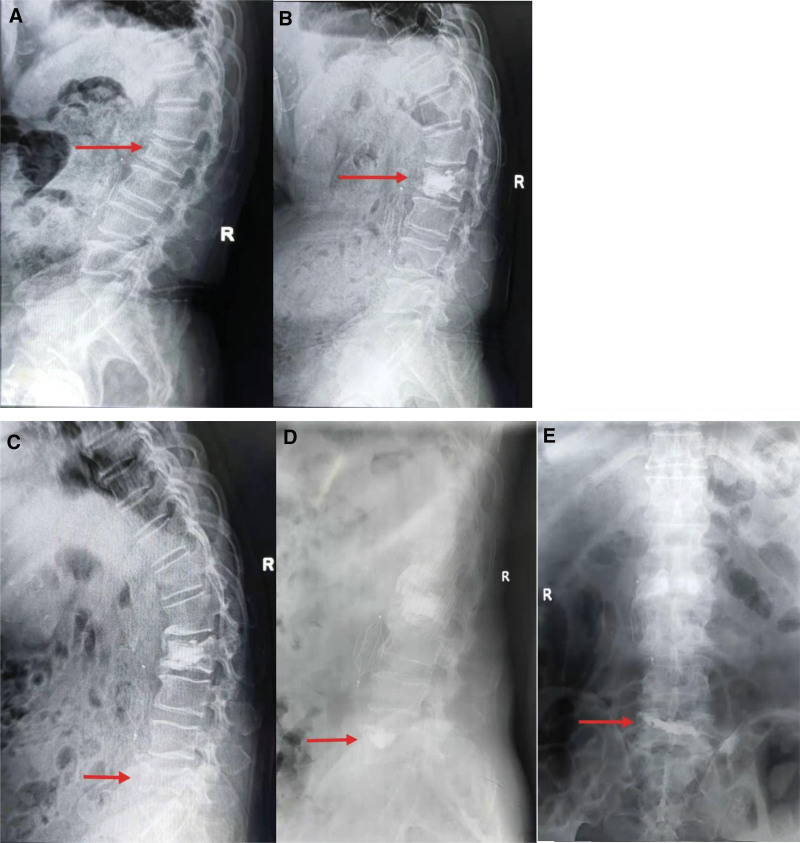
(A) A 72-year-old man with in L2 vertebral fracture presented with low back pain. (B) L2 was injected with bone cement to complete PKP. (C) Nineteen months after operation, the patient developed secondary vertebral fracture in L5. (D, E) The same operation was performed on L5 vertebral body. PKP = percutaneous kyphoplasty.

**Figure 6.**
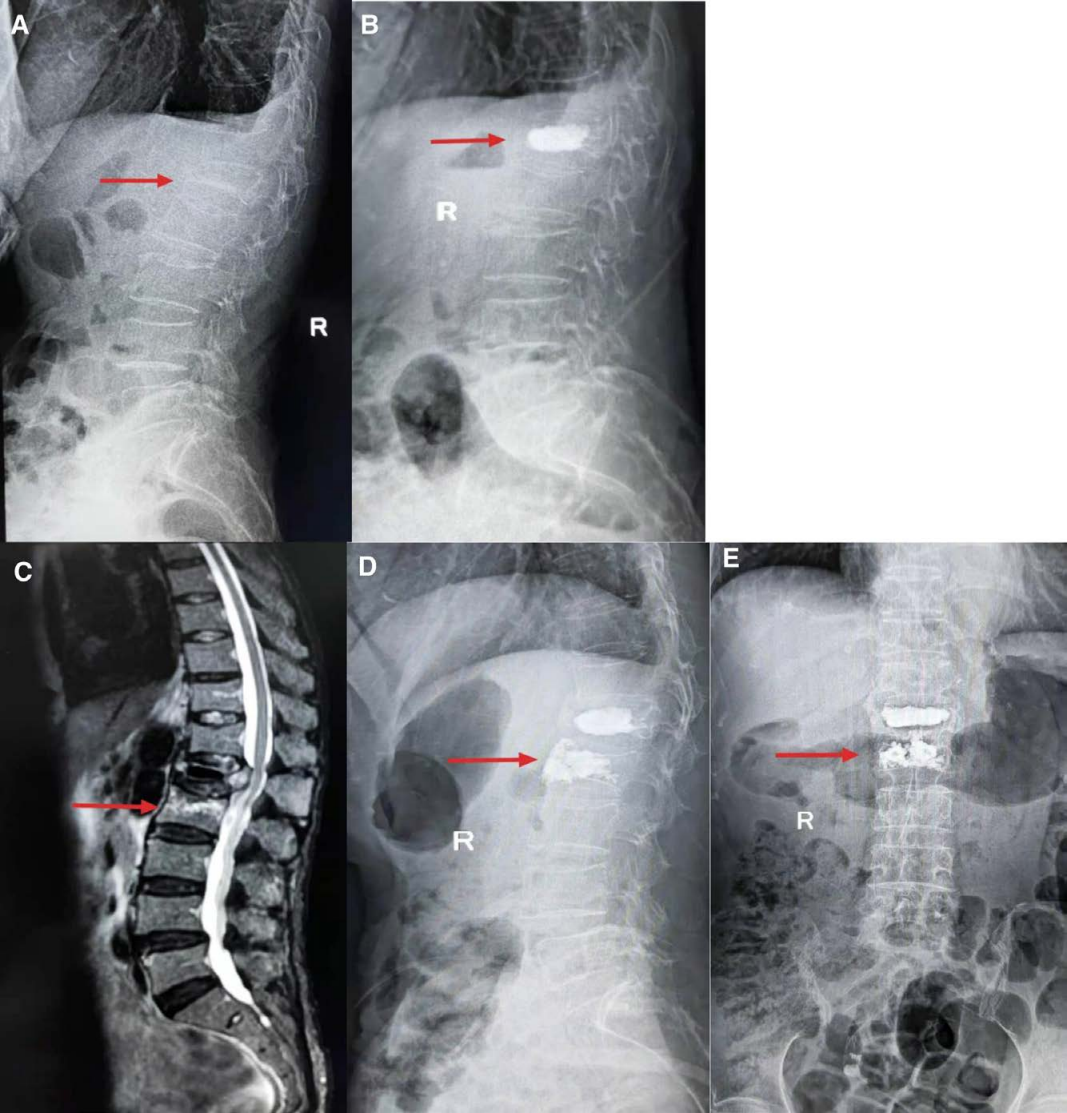
(A) A 73-year-old woman with in L1 vertebral fracture presented with low back pain. (B) L1 was injected with bone cement to complete PKP. (C) Eight months after operation, the patient developed secondary vertebral fracture in L2 (inferior adjacent vertebral body). (D, E) So, we performed the same operation on L2 vertebral body. PKP = percutaneous kyphoplasty.

Patients in both groups were followed up for an average of 22 months (3–45). We recorded secondary vertebral fractures throughout the follow-up period. In group Y new VCFs were observed among 5 patients (2.86%; Figs. 5 and 6), and 3 of the fractures were located in the adjacent vertebral body. But no secondary fracture was observed in group A. However, no statistical difference was identified between the 2 groups (Table 2).

**Table 2:** Complications.

	Refracture fracture	Well	
Group A	0	14	
Group Y	5	170	
Total	5	184	
*P* value			1.000

## 4. Discussion

When it comes to population, people often think of and talk about China, especially when talking about the elderly. China, as everyone knows, is the largest population in the world, and the proportion of the elderly is very large.^[12]^ The elderly are prone to suffer from many kinds of diseases, such as hypertension, diabetes, and osteoporosis, especially in those >90 years of age. Acute diseases, such as the severe coronavirus disease 2019, make the elderly unable to withstand the disaster. It is reported that more than two-thirds of the patients are >50 years old and have severe symptoms. Chronic diseases such as osteoporosis are systemic bone diseases characterized by decreased bone strength and changes in bone microstructure, resulting in an increased risk of spine and hip fractures. Spinal fractures caused by osteoporosis often occur in our institution. Our treatment of OVCFs patients has changed from PVP to PKP. However, some questions perplex us, such as the effect of minimally invasive treatment for fractures in patients aged 90 and over.

Kamei et al^[13]^ examined in detail the clinical effects of patients in their 90s receiving PVP, but did not observe the same-age patients receiving PKP. As far as we know, it is necessary to study the efficacy of PKP in patients aged 90 and over. Therefore, we evaluated the clinical effect and complication effect of patients treated with PKP. Both groups had unbearable pain before operation, and the difference between the 2 groups was not recognized. The postoperative pain was significantly relieved in both group Y and group A, which was different from that before treatment. We found that there was no difference in pain relief between group A and group Y, which demonstrated that PKP also had a significant effect on pain relief in elderly patients, which was consistent with the results reported in previous studies.^[14,15]^ The patient’s activity function was greatly reduced due to pain. After receiving PKP treatment, they can get out of bed under the protection of waist circumference, which not only improves the ability of daily activities and makes life unaffected but also reduces the complications caused by lying in bed, such as urinary system infection, lung infection, etc. There was no significant difference in this immediate improvement of motor function between group A and group Y, both before and after operation. Therefore, we believe that PKP treatment is urgently needed for elderly patients who need to improve pain and activity function.

Minimally invasive surgery can not only relieve pain and improve motor function, but also lead to disastrous complications, such as secondary vertebral fracture.^[16]^ In group Y new VCFs were observed among 5 patients (2.86%), but no secondary fracture was observed in group A. However, after statistical analysis, we found that no difference was revealed between group A and group Y. The incidence of secondary vertebral fracture after PKP treatment was 2.86%, less than 26% of the research report, significantly less than the previous maximum record value 50%.^[17,18]^ The reason for the significantly low rate of secondary vertebral fractures in this study is that our PKP operation method cleverly avoids the risk factors of new fractures. For example, we can achieve the effect of uniform distribution of bone cement in the vertebral body by using bilateral pedicle puncture, so as to reduce the risk of new vertebral fractures. Additionally, the patients included in this study were all low-risk people with new vertebral fractures, because patients with >1 fractured vertebral body and old vertebral fractures were excluded. We also found that 3 of fractured vertebral body were located in the adjacent vertebral body (60.00%), which is consistent with the results of Uppin et al’s^[19]^ study at the beginning of this century, although our results are smaller than his research values. New vertebral fractures are mainly located in adjacent vertebral bodies. We believe that adjacent vertebral fracture is caused by the high hardness of the vertebral body injected with bone cement and the mechanical load is transmitted to the adjacent vertebral body. This needs to be further confirmed because some scholars believe that adjacent vertebral fracture is only the natural progress of osteoporosis.^[20]^

We found no difference in clinical and complication indexes between group A and group Y. Interestingly, the basic characteristics of patients have transmitted discrepancy, such as bone mineral density, bleeding volume, and procedure time. Osteoporosis is often caused by bone mineral loss in the elderly. And bone mineral density is often used to quantify and evaluate osteoporosis. The bone loss in elderly patients aged 90 and above was significantly higher than that in young patients, especially in women, this phenomenon is more obvious. Postmenopausal women are in a state of oxidative stress due to lack of estrogen, which is an opportunity for osteoporosis.^[21]^ There is no doubt that our findings are consistent with the conclusion of Steiger et al’s study^[22]^ that age is inversely proportional to bone mineral density. Most patients aged 90 and over have cardiovascular diseases and have limited ability to tolerate surgery. Therefore, we shortened the operation time and completed it by experienced doctors on the premise of ensuring the accuracy of the operation. This is the reason for the identified difference in operation time between the 2 groups. Similarly, we found a significant reduction in bleeding volume while reducing the operation time. Although some scholars^[23]^ thought that the amount of intraoperative bleeding had nothing to do with age and gender, their view was not refuted by us because we reduced the operation time a lot, which caused this difference between the 2 groups.

There was no difference in follow-up time between the 2 groups, although group Y had a longer follow-up time. The original symptoms of low back pain were significantly relieved after operation, but what we are most worried about is the secondary vertebral fracture during follow-up, which will affect the daily life of patients again, and may shorten the life span of patients aged 90 and over. Before we carried out this study, no scholars had studied the safety of PKP treatment in patients aged 90 and over. Complications during the operation can cause death, such as pulmonary embolism caused by bone cement leakage, but such cases are rarely reported.^[24]^ Naturally, secondary vertebral fracture has become the main complication affecting the postoperative life of patients, especially for patients aged 90 and over. Fortunately, no secondary vertebral fractures were found in patients aged 90 and over during our follow-up. Although 5 patients in group Y were hospitalized due to unbearable low back pain again, no difference was identified between group A and group Y.

This study makes it clear that patients aged 90 and over can boldly accept PKP treatment, because there is no difference in pain relief, activity function improvement and long-term complications between them and younger patients. But some study limitations still exist in our study. To start with, first, this was a single-center retrospective study with a small number of cases and a short-term follow-up. Second, the evaluation of pain and motor function improvement lasted only 72 hours after operation.

## 5. Conclusion

Apparently, Our results show that PKP is safe and beneficial for patients aged 90 years or older.

## Author contributions

Bo Yang conceived the research design, Bo Yang collected data and papered the manuscript, Yangxue Zhao revised this article, Yangxue Zhao is responsible for this article.
